# Diagnostic Performance of Faecal Immunochemical Testing (FIT) in Patients with Lynch Syndrome Scheduled for Colonoscopic Surveillance

**DOI:** 10.3390/diagnostics14212431

**Published:** 2024-10-30

**Authors:** Adam D. Gerrard, Yasuko Maeda, Judith Strachan, Doug Speake, Malcolm G. Dunlop, Farhat V. N. Din

**Affiliations:** 1 Cancer Research UK Scotland Centre, Institute of Genetics and Cancer, University of Edinburgh, Edinburgh EH4 2XU, UK; 2 Department of Colorectal Surgery, Western General Hospital, Edinburgh EH4 2XU, UK; 3 School of Medicine, Dentistry and Nursing University of Glasgow, Glasgow G12 8QQ, UK; 4 Department of Surgery, Queen Elizabeth University Hospital, Glasgow G51 4TF, UK; 5 Blood Sciences, Ninewells Hospital and Medical School, Dundee DD1 9SY, UK; 6 UK Colon Cancer Genetics Group, Medical Research Council Human Genetics Unit, Medical Research Council Institute of Genetics & Cancer, Western General Hospital, The University of Edinburgh, Edinburgh EH4 2XU, UK

**Keywords:** Lynch syndrome, LS, faecal immunochemical testing, FIT, HNPCC

## Abstract

Background and Aims: Lynch syndrome (LS) carries a substantial lifetime risk of colorectal cancer which is currently mitigated by biennial colonoscopy surveillance. Paramount to the surveillance programme is the removal of adenomas before malignant transformation but there is an associated service burden and morbidity of repeated endoscopy. We investigated if faecal immunochemical testing (FIT) for faecal haemoglobin has the diagnostic performance to replace colonoscopy. Methods: In this retrospective cohort study, patients due to undergo planned surveillance for LS between November 2020 and April 2022 were sent two FIT kits prior to colonoscopy. Test diagnostic performance of colorectal cancer (CRC), advanced and non-advanced adenoma detection was calculated for single and double FIT strategies. A faecal-Hb of 10 µg Hb/g was considered positive. Results: In total, 78 patients, with 45 (57.7%) female, median age 52 years (IQR 41–63), completed at least one FIT and colonoscopy. The median time from FIT to colonoscopy was 47 days. A single FIT was positive in 7/30 cases of adenoma (2/3 advanced, 5/27 non-advanced). A total of 64 (82.1% of FIT1T returners) completed a second FIT. Using the greatest of the two FITs (FIT2TMAX) 8/26 (2/3 advanced, 4/23 non-advanced), patients with adenomas were identified. There were no cases of CRC. The sensitivity for adenoma detection was 23.3% and 23.1%, respectively. Conclusions: In patients with LS awaiting colonoscopy, FIT has a low sensitivity for detecting adenomas and advanced adenomas. This is not improved by the addition of a second FIT test.

## 1. Background

Lynch syndrome (LS) is the most common form of hereditary colorectal cancer (CRC), accounting for 1–3% of all CRCs [1]. It arises as a result of germline pathogenic variants in DNA mismatch repair (MMR) genes, most commonly MLH1, MSH2, MSH6, PMS2 or EPCAM, and carries a lifetime risk of CRC between 22 and 74% [2,3,4]. Due to the high CRC risk, regular colonoscopy is recommended every two years commencing at 25 or 35 years of age depending on the pathogenic gene, until age 75 [5,6]. Surveillance colonoscopies aim to detect early CRC development and prophylactically remove adenomas before they have the opportunity to progress. Patients with LS could therefore require 25 colonoscopies throughout their surveillance, acquiring cumulative morbidity risks. Whilst survival is improved through surveillance [7], up to 30% of CRCs associated with LS occur within the interval between planned endoscopy [8].

An objective, non-invasive method of assessing the risk of adenoma or CRC formation in patients with LS would be an advancement in current surveillance practice. The analysis of faecal haemoglobin using faecal immunochemical testing (FIT) is widely used in bowel cancer screening programmes and more recently has been positioned as the first line of investigation for patients with symptoms that may point to significant bowel disease, including colorectal cancer [9,10,11,12]. If the diagnostic performance of FIT in patients with LS was acceptable a two-stage surveillance programme could be implemented where a raised FIT triggered a colonoscopy and consequently reduced the number of procedures for patients.

We aimed to define the diagnostic performance of single and double FIT strategies for the detection of non-advanced adenomas, advanced adenomas and CRC in patients with LS at the time of their scheduled surveillance colonoscopy.

## 2. Methods

This was a retrospective cohort study of patients undergoing colonoscopy surveillance for LS within south-east Scotland. Inclusion criteria were patients with a confirmed pathogenetic variant of LS, or patients diagnosed with Lynch-like syndrome (LLS) by a clinical geneticist, due to colonoscopic surveillance. Patients with previous segmental colectomy were included as they require ongoing surveillance. Exclusion criteria were patients with previous a subtotal colectomy or panproctocolectomy, or patients outside the age range of recommended surveillance [5]. As part of the endoscopy recovery pathway following COVID-19, all patients on endoscopy waiting lists received FIT to aid list prioritisation. Within NHS Lothian, it is routine clinical practice to perform two FIT tests several days apart. The results of these FIT tests were compared against subsequent endoscopic findings.

Identified patients were sent the first FIT kit when they were three months from their due endoscopy. The second FIT was requested upon the return of the previous FIT. Prior to commencing bowel preparation, patients were asked to return each kit once completed to their local primary care centre where they were then transported to the UKAS-accredited NHS Tayside Blood Sciences laboratory based in Ninewells Hospital, Dundee, in a timely fashion, where samples were analysed to ISO15189 standards. There was no medication or dietary restrictions prior to completing the FIT.

### 2.1. FIT

FIT kits (Minaris Medical Co., Ltd., Tokyo, Japan) were mailed to patients with instructions on how to perform the test. Samples were returned to the UKAS-accredited NHS Tayside Blood Sciences laboratory based in Ninewells Hospital (Dundee) where samples were analysed to ISO15189 standards using the HM-JACKarc analyser (Minaris Medical Co., Ltd., Tokyo, Japan) which has a reporting limit of quantification (LoQ) of 7 µg Hb/g and a maximum reported result of 400 µg Hb/g. The test positivity threshold was f-Hb of 10 µg Hb/g.

### 2.2. Data Collection

Patient demographics, pathogenic gene variant and date of planned colonoscopy surveillance were prospectively collected. FIT values (µg Hb/g) and test dates were recorded. Colonoscopy findings were categorised as CRC, advanced adenoma (polyp with high grade dysplasia, ≥10 mm with low grade dysplasia or sessile serrated lesions with any dysplasia), non-advanced adenoma (adenoma without advanced features) or no significant bowel pathology (no CRC, advanced or non-advanced adenoma).

### 2.3. Data Analysis

Data analysis was performed using R v4.0.5 [13] with associated packages and GraphPad Prism Version 10.3.1 [14]. Diagnostic accuracy for positive predictive value (PPV), negative predictive value (NPV), sensitivity and specificity were calculated with 95% confidence intervals. The Scottish Index of Multiple Deprivation (SIMD) tool was used to assess levels of socio-economic deprivation; this creates decile ranks from the most (1) to least (10) deprived areas from postcodes. Categorical data were compared by Fisher’s exact or Chi-squared test as appropriate and continuous data by *t* test or Mann–Whitney U tests. Receiver Operating Characteristic (ROC) curves were generated for the first FIT result (FIT1T), the greatest FIT result in those to complete both FITS (FIT2TMAX) and for the greatest, or only, FIT of any returned (FITANYMAX). This work formed part of routine clinical practice and local Caldicott approval (#20154) was granted.

## 3. Results

**Figure 1:**
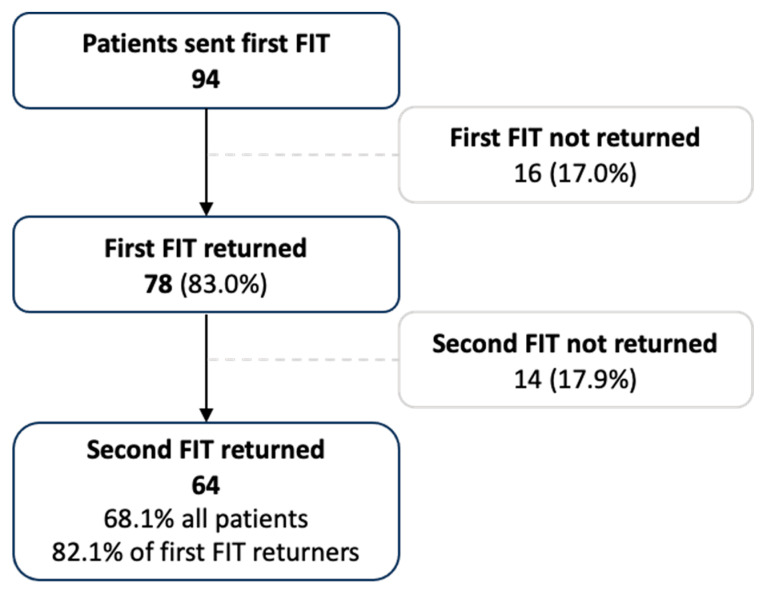
Study flow diagram of the number of patients who were sent the first FIT, returned this first FIT and subsequently sent and returned the second FIT prior to colonoscopy.

Between November 2020 and April 2022, 94 patients who were due to undergo surveillance endoscopy were sent their first FIT; 78 (83.0%) completed at least one FIT prior to colonoscopy (Figure 1).

Of the 78 patients that returned one FIT, the median age was 52 years, 45 (57.7%) were female and 16 had undergone a previous segmental colectomy (Table 1). For nine patients, this was their first surveillance. In the 69 patients undergoing repeat surveillance, the median time between endoscopies was 27 months (IQR 25–30) and 15 (23.1%) had adenomas during their previous endoscopy. The median time between FIT1 and FIT2 was 16 days (IQR 9–22). The median time from FIT completion to investigation was 47 days (IQR 17–82). At colonoscopy, 30 patients were diagnosed with adenomas (27 non-advanced, 3 advanced) and there were no CRCs.

### 3.1. Single FIT Approach

The utility of a one test strategy in patients with LS was investigated using the result of the first FIT to be performed (FIT1T). The positivity rate of FIT1 was 11.5%. The three cases of advanced adenoma had FITs 0, 38, and 63 (Table 2). Of the non-advanced adenomas, 22 of 27 had a FIT1T less than 10 µg Hb/g, resulting in the overall sensitivity of one FIT for any adenoma (advanced and non-advanced) being 23.3% (Table 3). A positive FIT1 was associated with the finding of an adenoma (*p* = 0.02), which showed a trend towards previous segmental colectomy (*p* = 0.08) but was not affected by previous polyp burden, age, or sex (Table 4). Of the two positive FITs with no adenoma pathology, one had haemorrhoids whilst the other had a normal scope but had a history of previous segmental colectomy. There were no complications reported from the colonoscopies performed.

The sixteen patients not to complete a FIT had a trend in being younger than those who returned a test (median age 40 vs. 52 *p* = 0.056). All were investigated at a median 28.5 months (IQR 26–31) from previous colonoscopy. Three non-advanced adenomas were found.

### 3.2. Multiple FIT Approach

**Figure 2:**
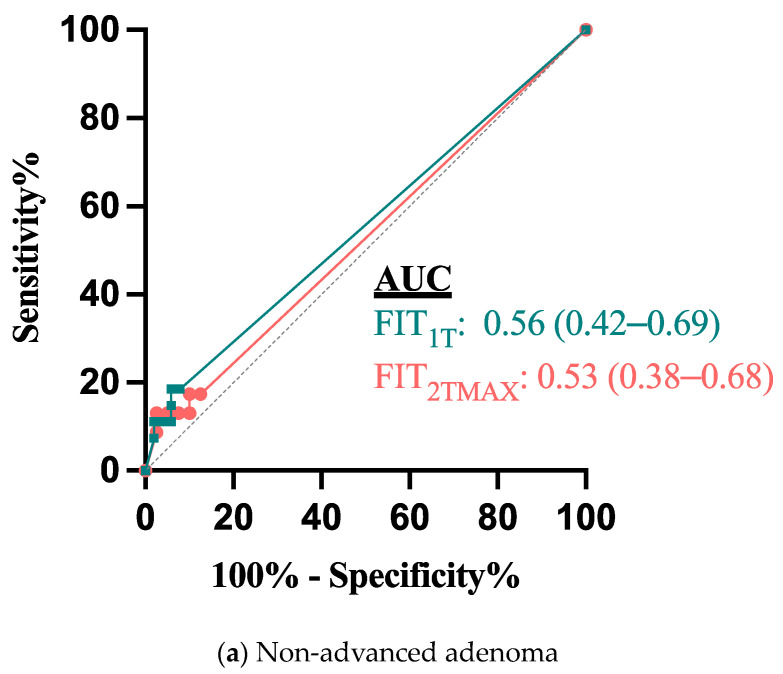

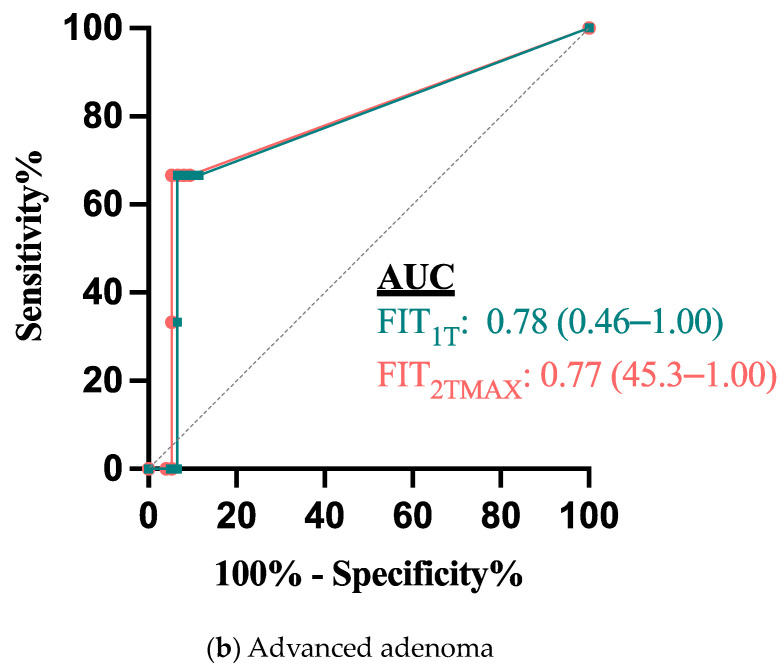
ROC curve analysis for (**a**) non-advanced and (**b**) advanced adenoma detection by FIT1T and FIT2TMAX. FIT1; first FIT performed by a patient, FITMAX; greatest FIT result of each patient: (**a**) non-advanced adenoma; (**b**) advanced adenoma.

In total, 64 (82.1%) of all of the included patients returned a second FIT (FIT2). The positivity rate of any of the two FITs being over 10 µg Hb/g was 14.1%, with no difference in positivity between the first and second FIT (12.5% v 7.8%, *p* = 0.56). The addition of the second FIT did not improve the diagnostic detection of advanced or non-advanced adenomas. ROC analysis showed no improvement to the area under the curve (AUC) by FIT2TMAX compared to FIT1T (Figure 2).

The variation between multiple test results for individual patients was assessed. Discordance (one test positive, one negative or vice versa) occurred in patients with LS both in the presence and absence of pathology. Of the 64 to complete two tests, 5 (7.8%) had discordant results, and of the 26 with an adenoma, 2 (7.7%) had one test positive and another negative at the threshold of 10 µg Hb/g.

There was no difference in the time to colonoscopy for patients, regardless of FIT result.

## 4. Discussion

The diagnostic performance of FIT in this study for detecting advanced and non-advanced colorectal adenomas is not sufficiently high enough to advocate its use as a screening route to colonoscopy in the surveillance programme for LS. In fact, 23/30 (76.7%) and 20/26 (76.9%) adenomas would be missed by a single and FIT2TMAX strategy. FIT has been successfully incorporated into clinical guidelines for significant bowel disease detection in symptomatic patients [11], but even in these symptomatic cohorts, sensitivity for advanced adenomas only ranges between 24 and 51% [15,16]. This is not surprising given FIT is a test for blood, and it is presumed that many adenomas do not bleed in contrast to CRCs [17].

Fundamentally, the difference in FIT adoption between symptomatic patients and LS surveillance is the pathology being targeted. In symptomatic cohorts, FIT is used to risk stratify and detect cases of likely CRC and other significant bowel diseases. In LS surveillance, the emphasis is on detecting and removing adenomas to reduce the risk of malignant development, which is accelerated compared with the general population [18,19]. FIT does not detect advanced adenomas and early-stage CRC effectively [20,21,22]. A meta-analysis estimated the sensitivity of FIT for T-stage 1 CRC to be 40% (21–64%) [21]. It is thought that some adenomas and even early-stage CRC do not bleed, either in high enough quantities or persistently enough, for there to be a substantial amount of haemoglobin within the sampled stool to generate a positive FIT result. Previous work from our group reported the sensitivity of FIT, at a 10 µg Hb/g threshold, for the detection of advanced adenomas to be only 51.4%. Furthermore, when the FIT test was repeated, 20.5% of patients with a CRC or advanced adenomas had discordant FIT results (one positive and one negative, or vice versa) [16]. Therefore, relying on a positive FIT to trigger surveillance colonoscopy would risk the diagnosis of these pre-malignant or early CRCs being delayed, resulting in more advanced malignancies and rendering the surveillance programme futile. Furthermore, the relationship between previous segmental colonic resection and a positive FIT means the test may not be suitable for such patients as this may result in an increased number of colonoscopies for these patients. Further larger studies are needed to conclude if this is a significant relationship.

There was a satisfactory return rate for FITs, indicating that multiple testing is acceptable to patients with LS undergoing surveillance. The overall 7.8% discordance in repeat testing may show a future utility of a multiple testing strategy, although this must be balanced against the increase in positivity which did not lead to increased adenoma detection in this study. The reported percentage improvement for advanced adenoma detection by double FIT is between 14.2 and 50.0% [15,23,24,25,26,27,28,29]. Previously, FIT in LS was studied as a subgroup analysis of 17 patients where one advanced adenoma was detected by FIT and one non-advanced adenoma was not [30]. Run contemporaneously with our study, a further study has investigated the use of a single FIT for risk stratifying urgent colonoscopy in patients with LS during the COVID-19 pandemic [31]. Here, 339/558 patients (68.8%) completed a FIT with a 17.7% positivity rate at a 10 µg Hb/g threshold. Similar to the findings in our study, the sensitivity for detecting advanced colorectal neoplasia was found to be 64.7%, with patients returning a positive FIT being prioritised for colonoscopy over those with FIT less than 10 µg Hb/g, with a different median time from the test result (49 vs. 122 days).

Other non-invasive strategies to detect adenomas have been suggested. In LS, magnetic resonance and computed tomography colonography have been shown to detect larger (≥1 cm) polyps with a 60–100% sensitivity but have similar limitations to FIT for smaller polyps, with a sensitivity between 25 and 50% [32,33]. Stool RNA tests offer further methods of detecting colonic lesions; however, these are also beset by the same problem that whilst sensitivity for CRC is high (95%), the detection of smaller non-advanced adenomas remains too low (25%) to be used in the context of LS surveillance [34]. Polygenic risk scores have been proposed as a means to risk stratify CRC and advanced colorectal neoplasia in the setting of bowel screening and symptomatic patients [35,36]; however, these have not been powered to detect smaller adenomas or validated within the LS population.

A limitation to this study is sample size; however, the known study population is relatively rare. LS is thought to have a prevalence of 0.3% in the UK but only 5% of these are identified cases [37]. FIT kits were sent to patients who were due their surveillance and because of this, the data only reflect FIT results prior to planned surveillance and the use of FIT in interval years cannot be assessed. Nevertheless, this is the first study to aim to systematically assess single and multiple FIT strategies in patients with LS. Future studies should continue to explore the benefits of single and multiple FIT in LS, particularly within the interval year of screening. A large multicentre study of single FIT is currently in the recruitment stage, aiming to answer these questions [38].

The low sensitivity for advanced and non-advanced adenomas makes positioning FIT as a gatekeeper to colonoscopy surveillance unappealing. Within the scope of this study, FIT cannot replace the current practice of biannual colonoscopic surveillance. However, FIT may have a place in situations where demand for colonoscopy results in the surveillance period becoming overdue. Here, a positive FIT could prioritise a person’s surveillance over a negative test.

## Figures and Tables

**Table 1:** Characteristics of patients included in the study. Patient demographics and previous surgical history of the included patients. Positivity rate (≥10 µg Hb/g) was calculated for the first (FIT1T), greatest of two completed tests (FIT2TMAX) and greatest, or only FIT, of all returners (FITANYMAX).

Characteristic	
Number of patients	78
Sex, female (%)	45 (57.7)
Median age (IQR)	52 (41–63)
Gene with pathogenic variant	
MSH6	28 (35.9)
MLH1	23 (29.5)
MSH2	20 (25.6)
PMS2	2 (2.6)
EPCAM	1 (1.3)
LLS	4 (5.1)
Previous segmental colectomy (%)	16 (20.5)
Right hemicolectomy	8 (50.0)
Anterior resection	4 (25.0)
Abdominal perineal resection	3 (18.8)
Hartmann’s procedure	1 (6.2)
Adenomas at previous colonoscopy (%)	15 (23.1)
Median time form FIT to colonoscopy (days, IQR)	47 (17–82)
Positivity rate	
FIT_1T_	11.5%
FIT_2TMAX_	14.1%
FIT_ANYMAX_	12.8%
Colonoscopy findings (%)	
No significant bowel pathology	48 (61.5)
Non-advanced adenoma	27 (34.6)
Advanced adenoma	3 (3.8)

**Table 2:** Characteristics and FIT results of patients with bowel pathology.

Pathology	Sex	Age	Pathogenic Variant	FIT Results (µg Hb/g)
Advanced adenoma				
	F	34	MLH1	0, 0
	M	37	MLH1	63, 89
	F	67	MSH2	38, 26
Non-advanced adenoma				
	F	35	MLH1	0, 0
	M	35	MLH1	0
	F	35	EPCAM	13, 0
	F	41	MLH1	0, 0
	F	41	MSH6	0
	F	42	MLH1	0
	M	43	MSH6	0, 0
	M	43	MLH1	0, 0
	M	46	MSH6	400, 10
	M	47	MSH6	0, 0
	F	49	MSH6	0, 0
	M	52	MSH2	0, 0
	M	53	MLH1	400, 0
	F	55	MSH6	0, 0
	M	56	MLH1	0, 0
	F	56	MSH2	0, 0
	M	58	MSH6	0, 0
	M	58	MSH6	0, 0
	F	64	MSH6	0, 0
	F	66	LLS	0, 0
	M	69	MSH2	163, 23
	F	69	MSH6	0, 0
	M	70	MLH1	32
	F	74	MSH2	0, 0
	M	75	MLH1	0, 0
	F	76	MSH6	0, 0
	M	77	MLH1	0, 0

**Table 3:** Test performance of FIT1T and FIT2TMAX for advanced adenoma and non-advanced adenoma detection. FIT1T; first FIT performed by a patient, FIT2TMAX; greatest FIT result of patients to complete two tests.

	*n*	Sensitivity	Specificity	NPV	PPV
FIT_1T_					
All adenoma	30	23.3 (1.9–42.3)	95.8 (85.7–99.5)	66.7 (54.3–78.1)	77.7 (40.0–97.2)
Advanced adenoma	3	66.7 (9.4–99.2)	90.7 (81.7–96.2)	98.6 (92.2–99.9)	22.2 (2.8–60.0)
Non-advanced adenoma	27	18.5 (6.3–38.1)	92.2 (81.1–97.8)	68.1 (55.8–78.8)	55.6 (21.2–86.3)
FIT_2TMAX_					
All adenoma	26	23.1 (9.0–43.6)	92.1 (78.6–98.3)	63.6 (49.6–76.2)	66.7 (29.9–92.5)
Advanced adenoma	3	66.7 (9.4–99.2)	88.5 (77.8–95.3)	98.2 (90.3–99.9)	22.2 (2.8–60.0)
Non-advanced adenoma	23	17.4 (5.0–38.8)	87.8 (73.8–95.9)	65.5 (51.4–77.8)	44.4 (13.7–78.8)

**Table 4:** Comparison of patients with a positive or negative FIT result. FIT1; first FIT performed by a patient, FIT2TMAX; greatest FIT result of patients to complete two tests.

**FIT_1T_**		**Negative**	**Positive**	***p*-Value**
	Number of patients (%)	69	9 (11.5)	
	Sex, F (%)	42 (60.9)	3 (33.3)	0.16
	Median age (IQR)	52 (41–61)	53 (41–69)	0.58
	Previous segmental colectomy (%)	12 (17.4)	4 (44.4)	0.08
	Previous adenomas (%)	12 (20.3)	3 (37.5)	0.36
	Adenoma diagnosed	23 (33.3)	7 (77.8)	0.02
**FIT_2TMAX_**		**Negative**	**Positive**	***p*-Value**
	Number of patients (%)	55	9 (14.1)	
	Sex, F (%)	35 (63.6)	3 (33.3)	0.14
	Median age (IQR)	54 (44–64)	46 (37–68)	0.52
	Previous segmental colectomy (%)	9 (16.4)	3 (33.3)	0.35
	Previous adenomas (%)	11 (20.0)	3 (33.3)	0.40
	Adenoma diagnosed	20 (36.4)	6 (66.7)	0.14

## Data Availability

Summarised anonymised data will be made available on request.
